# IgG4 related disease as a cause of isolated retroperitoneal fibrosis with no other organ involvement; Case report

**DOI:** 10.1016/j.amsu.2020.12.028

**Published:** 2020-12-23

**Authors:** Almurtada Razok, Rubab Malik, Priyanka Cackamvalli, Muhammad Zahid

**Affiliations:** a Department of Internal Medicine, Hamad Medical Corporation, P.O 3050, Doha, Qatar; b Department of Rheumatology, Hamad Medical Corporation, P.O 3050, Doha, Qatar

**Keywords:** IgG4 related disease, Retroperitoneal fibrosis, Rheumatology, Histopathology

## Abstract

**Introduction and importance:**

Retroperitoneal fibrosis (RPF) is a rare disease characterized by the replacement of normal tissue with fibrosis and inflammation. We present the case of a 40-years-old gentleman with RPF whose presenting complaints were bilateral flank pain and weight loss and was found to have IgG4 related disease. To the best of our knowledge, IgG4-related disease with isolated retroperitoneal involvement is a very rare occurrence.

**Case presentation:**

The diagnosis of IgG4-Related retroperitoneal fibrosis was made based on clinical, radiological and histopathological criteria. Imaging revealed isolated retroperitoneal involvement and the patient was started on oral steroids with a good clinical response after ten days. Repeated imaging months later showed significant regression in the fibrosis.

**Clinical discussion:**

RPF can occur due to many etiologies and is categorized to idiopathic and secondary. Factors associated with secondary RPF include medications, autoimmune disease, malignancy and IgG4 related disease. Almost all the reported cases of IgG4 related disease had evidence of multifocal involvement such as the pancreas, aorta and kidneys. Our patient was diagnosed with isolated RPF due to IgG4 disease. He met all the suggested diagnostic criteria, was started on oral steroids and had an excellent clinical outcome.

**Conclusion:**

IgG4 related disease can present with isolated retroperitoneal fibrosis without involvement of other organ systems. The diagnosis should be based on specific criteria. Treatment with corticosteroids can lead to remission both clinically and radiographically.

## Highlights

• Retroperitoneal fibrosis can present in a variety of clinical pictures.
• IgG4 related disease can present with isolated retroperitoneal fibrosis.
• It is diagnosed based on clinical, radiological and pathological criteria.
• Treatment with corticosteroids can lead to clinical and radiological remission.

## 1 Introduction

Retroperitoneal fibrosis (RPF) is a condition that was initially described as chronic periaortic inflammation [1]. It is a rare disease characterized by the development of inflammation and fibrosis in the soft tissues of the retroperitoneum and abdominal organs [2]. First reported case was in 1905 by a French urologist who described ureteric obstruction as a consequence [3]. Retroperitoneal fibrosis is generally categorized to idiopathic retroperitoneal fibrosis (IRPF) which represents two third of the cases, and secondary retroperitoneal fibrosis, which is due to several triggers such as infections, radiographic exposure, medications, autoimmune disease, IgG4 related disease and malignancy [4]. We report this case to highlight IgG4 related disease as a cause of retroperitoneal fibrosis. As IgG4 related disease is a steroid-responsive pathological process, diagnosing it can prevent the exposure of patients with retroperitoneal fibrosis to unnecessary diagnostic and therapeutic interventions. Not only is retroperitoneal fibrosis due to IgG4 related disease rare, but what makes this case unique is that the patient did not have any evidence of involvement in other parts of his body which is a very rare occurrence.

## 2 Presentation of case

A 40-years-old gentleman with no previous medical history, presented to the hospital via ambulance with chief complaints of flank pain and weight loss. The patient was a non-smoker and non-alcoholic and was not taking any medications at home. He worked as a driver for the last two years. He was not sexually active, and there was no recent travel abroad. Family history was negative. The patient reported bilateral flank pain for six months duration that was mild to moderate in intensity, dull in nature and most noticeable during the night. There was weight loss of 14 kg in four months. Vital signs were normal and physical examination was positive for mild tenderness in the flank region bilaterally. Detailed examination of other systems, including the lymphatic was normal. Blood investigations including complete blood count, comprehensive metabolic panel, erythrocyte sedimentation rate, C - reactive protein (CRP), serum and urine protein electrophoresis, thyroid function tests, serum Quantiferon and full autoimmune panel including rheumatoid factor, antinuclear, ribonucleoprotein (ANA), smith and Scl-70 antibodies were negative. HIV and hepatitis serology were also negative. Serum immunoglobulin measurement was positive for elevation in total IgE with a level of 1510 kunits/L (reference range between 0 and 114) and in IgG4 (204 mg/dL with reference range of 3–201).

**Fig. 1:**
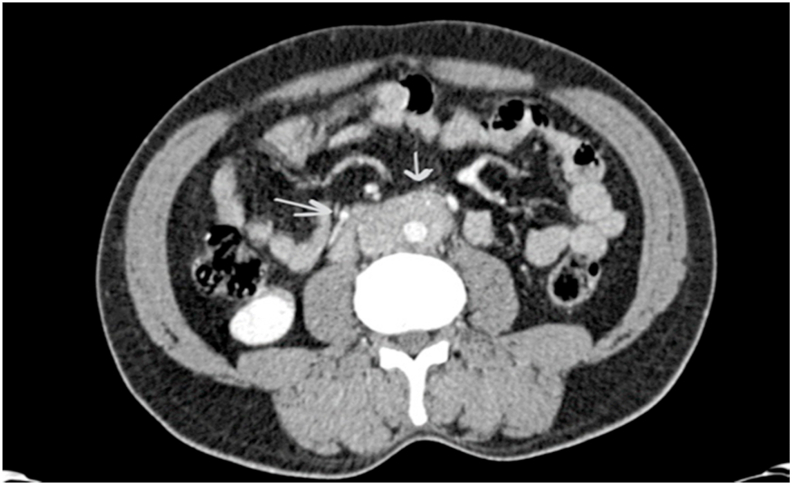
CT scan of the abdomen showing Retroperitoneal soft tissue density encasing the abdominal aorta, Inferior vena cava and common iliac arteries. (This figure does not need to be printed in color).

Computed tomography scan of the chest, abdomen and pelvis with contrast revealed a retroperitoneal soft tissue mass with vascular encasement of the abdominal aorta and inferior vena cava with no ureteric involvement (Fig. 1).

**Fig. 2:**
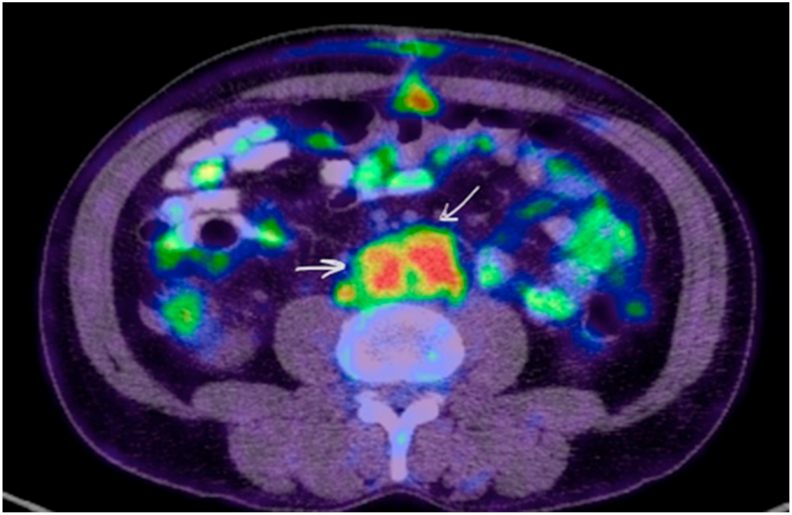
FDG Whole body PET CT showing Retroperitoneal mass lesion encasing the aorta, inferior vena cava and common iliac arteries with hypermetabolic activity (SUV max 9.1). (This figure does not need to be printed in color). (For interpretation of the references to color in this figure legend, the reader is referred to the Web version of this article.)

Whole body positron emission tomography (PET) scan revealed the same retroperitoneal soft tissue mass with hypermetabolic activity and no increased fluorodeoxyglucose uptake in other parts of the body (Fig. 2).

**Fig. 3:**
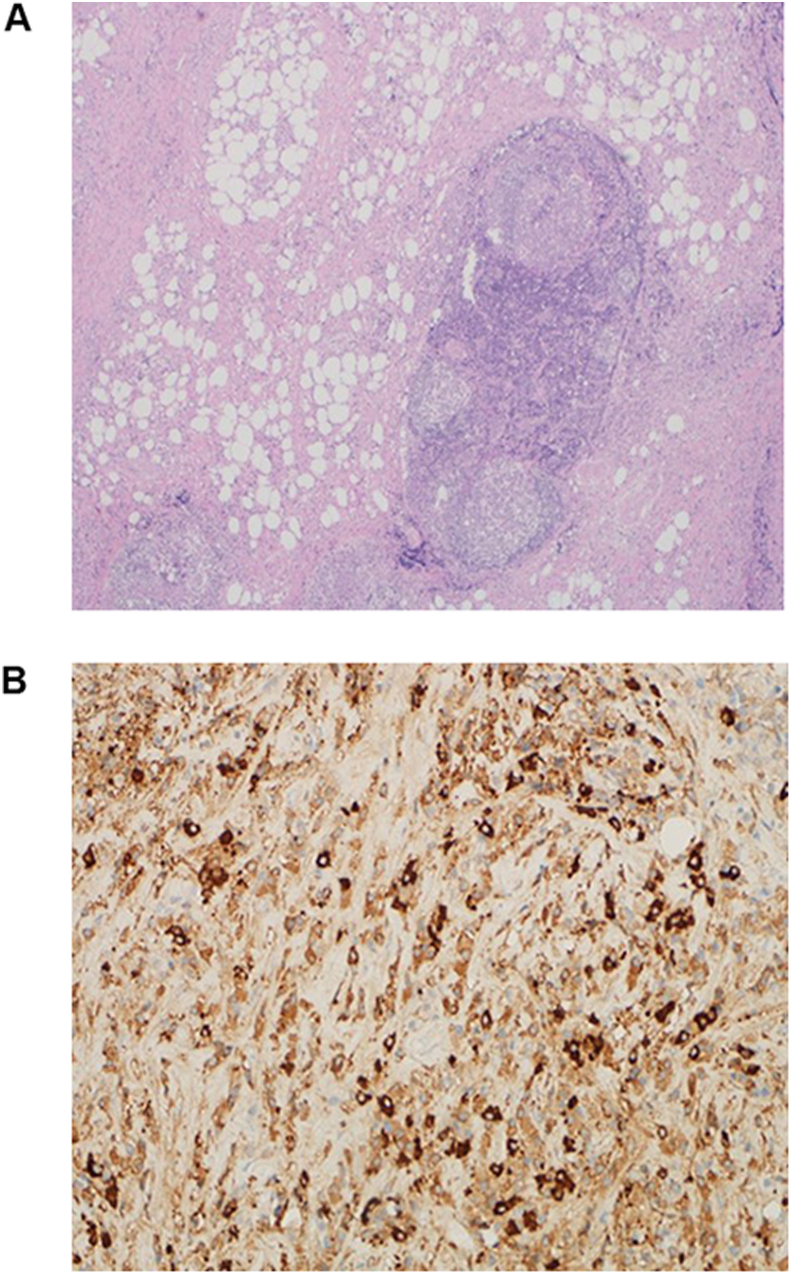
Histopathology figures showing dense collagen fibers infiltrated by dense mixed chronic inflammation (3a). Immunostains showing increased number of IgG4 positive plasma cells that are more than 10/HPF, about 35/HPF in most of the involved areas (3b). (This figure does not need to be printed in color).

The general surgery team performed an open laparotomy biopsy after explaining the risks of possible vascular injury to the patient. The procedure was performed by a senior consultant in general surgery, was assisted by an associate consultant in acute care surgery and was uneventful. The biopsy revealed dense collagen fibers infiltrated by dense mixed chronic inflammation (Fig. 3a). The inflammatory infiltrate was composed mainly of plasma cells, with great tendency of perivascular arrangement. Widespread obliterative endophlebitis was also present. There was no evidence of atypical spindled, epithelioid, Reed Sternberg or lymphoid cells. There was no evidence of necrosis or malignancy. Ancillary studies revealed increased number of IgG4 positive plasma cells (more than 40% with 35 cells/HPF in most of the involved areas) (Fig. 3b).

He was started on oral prednisolone 40 mg per day and discharged home. The patient was followed in the outpatient department of the hospital. He was seen in the rheumatology clinic three times post-discharge: at ten days, at three months and at six months. The patient was inquired about the intensity of his pain based on the numeric rating scale (1–10) and was significantly lower at all three visits compared with his status prior to starting steroids. Repeated labs after three and six months showed a considerable reduction in IgG4 levels (44.3 and 37.4 mg/dL respectively). CT scan of the abdomen and pelvis with contrast at six months post-discharge revealed significant regression of the retroperitoneal fibrosis. He received a tapering course of prednisolone for a total duration of three months. He confirmed daily compliance to the medication and did not report any side effects.

## 3 Discussion

IgG4 related retroperitoneal fibrosis is usually part of a systemic process characterized by fibro-inflammatory changes that can manifest as mass-like lesions in the retroperitoneal space [5]. Clinical symptoms are nonspecific and include abdominal pain, back pain, and edema of the lower extremities [6]. IgG4-related RPF has a good response to therapy with steroids; therefore, early identification and treatment reduce the rate of unnecessary therapeutic modalities [7].

IgG4 related systemic disease is an increasingly recognized entity in the last decade [8]. IgG4 - RPF is more common in males with a ratio of 3.3:1 and average age at diagnosis of 66 years [9,10]. It is a fibro-inflammatory disorder that is associated with infiltration of various organs by IgG4 positive plasma cells [11]. Organs which can be involved include the kidneys, ureters, prostate, thoracic and abdominal aorta, pancreas and retroperitoneal space [12]. Clinical manifestations include pancreatitis, cholangitis and sialadenitis [13]. The gold standard for asserting the diagnosis is biopsy of the involved organ, with pathological confirmation based on the fraction of IgG4 positive plasma cells and the pattern of infiltration [11]. Suggested criteria for the diagnosis include diffuse or local involvement of one or more organs, elevated IgG4 serum levels of more than 135 mg/dl, and on histopathology, more than 40% IgG4/IgG positive plasma cells ratio with more than 10 IgG4 positive cells per high power field [14]. Elevated serum IgG4 supports the diagnosis, although normal levels do not exclude it [15]. Our patient had nonspecific symptoms of bilateral flank pain and weight loss, imaging showed mass encasing aorta and inferior vena cava. IgG4 was raised at 204 mg/dl and histopathology showed more than 40% IgG positive plasma cells in the involved areas.

In an analysis of 40 patients with retroperitoneal fibrosis, it was found that IgG4-RPF occurred more commonly in older males with the majority of the patients having extra-retroperitoneal involvement. On the other hand, non IgG4 related RPF was more common in younger females and the disease was mainly confined to the retroperitoneal space. Both conditions had an adequate response to steroids [3,16].

Retroperitoneal fibrosis due to IgG4 related disease was reported before, however almost all of the reported cases had evidence of multifocal involvement including pituitary, salivary glands, lymph node, mediastinum and pancreas [11]. To the best of our knowledge, isolated retroperitoneal fibrosis due to IgG4 related disease without other organ involvement is a very rare occurrence.

## 4 Conclusion and learning points

IgG4 related disease can present with isolated retroperitoneal fibrosis without involvement of other organ systems. The diagnosis should be confirmed based on clinical, radiological and histopathological criteria. Treatment with corticosteroids and close follow up can lead to excellent outcomes.

This work was reported in line with the CARE guidelines.

## Patient consent

Written informed consent was obtained from the patient for publication of this case report and accompanying images. A copy of the written consent is available for review by the Editor-in-Chief of this journal on request.

## Patient perspective

Although I had a long stay in the hospital and had to go through multiple investigations, I was pleased that the medical team were able to identify my diagnosis and provide me with the appropriate treatment

## Funding statement

This article did not receive any specific grant from funding agencies in the public, commercial, or not-for-profit sectors.

## Declarations of competing interest

None to be declared.

## Author contribution statement

AR and RM performed literature review and wrote the original draft of the manuscript. PC and MZ supervised the writing process. All authors approved the final version for submission.

## Registration of Research Studies

Not applicable.

Our manuscript is a case report.
